# Bioprotective Potential of Lactic Acid Bacteria in Pickled Pepper Rabbit Meat During Refrigerated Storage

**DOI:** 10.3390/foods14162918

**Published:** 2025-08-21

**Authors:** Jiamin Liang, Bo Wang, Jiamin Zhang, Ting Bai, Zhenguo Zhong, Zhonghua Tang

**Affiliations:** 1 Meat Processing Key Laboratory of Sichuan Province, Chengdu University, Chengdu 610106, China; ljm13672537719@126.com (J.L.); wangbo9214@163.com (B.W.); baiting13@163.com (T.B.); 2 BaHang Food Development Co., Zigong 643000, China; 18719300385@163.com; 3 SiChuan AiChiTu Food Co., Bazhong 636600, China; 13672537719@163.com

**Keywords:** fermented meats, microbial succession, quality control, *Latilactobacillus curvatus*, storage property

## Abstract

The impacts of *Lactilactilactobacillus sakei* (LS), *Pediococcus acidilactici* (PA), and *Latilactobacillus curvatus* (LC) on quality properties, protein and lipid oxidation, and microbial dynamics of pickled pepper rabbit meat during refrigerated storage (4 °C for 1, 3, 5, and 7 days) were investigated. The results showed that the addition of lactic acid bacteria bioprotective agents effectively reduced the pH of pickled pepper rabbit meat, inhibited protein and lipid oxidation, suppressed the growth and proliferation of spoilage bacteria, and maintained favorable textural characteristics. Among the tested strains, *Latilactobacillus curvatus* exhibited the most significant preservation effects throughout the storage period. On day 7 of storage, the TBARS value of the LC group was 20.60% lower than that of the LS group and 14.68% lower than that of the PA group. Similarly, the total carbonyl content was 12.30% lower than the LS group and 6.21% lower than the PA group, while the total sulfhydryl content was 20.81% and 10.12% higher, respectively. Additionally, the TVB-N value was 11.91% lower than the LS group and 4.37% lower than the PA group. Additionally, the *Latilactobacillus curvatus* group maintained a lower pH, superior elasticity, chewiness, and cohesiveness, while effectively inhibiting spoilage bacterial growth and proliferation. In conclusion, *Latilactobacillus curvatus* was the most effective bioprotective agent for preserving the storage characteristics of pickled pepper rabbit meat.

## 1. Introduction

Rabbit meat is highly favored by consumers due to its low fat, low cholesterol, and high protein content and it has delicate muscle fibers and collagen-rich connective tissue [1]. In recent years, Pre-cooked and ready-to-consume rabbit meat products are being gradually introduced into the market. Pickled pepper rabbit meat serves as a representative of such ready-to-eat products. The unique pickled pepper flavor, combined with the collagen-rich connective tissue in pickled pepper rabbit meat, imparts to it a distinct texture featuring tenderness and elasticity. Consequently, this product is attracting growing favor among consumers, and is similar to other pickled meat products, such as pickled pepper chicken feet, which are priced around three times higher than ordinary meat products. Pickled pepper rabbit meat not only offers a distinctive flavor experience but also holds high added value and significant market potential [2].

During the processes of transportation and storage, pickled pepper rabbit meat is prone to quality degradation. Microbial contamination stands as a primary determinant influencing the product quality. Meat products are highly perishable due to the potential growth of pathogenic bacteria such as Listeria monocytogenes, *Staphylococcus* aureus, and *Pseudomonas* spp., posing significant food safety risks. Traditional physical sterilization approaches, such as heat treatment, have the potential to disrupt the collagen fiber structure within rabbit meat, resulting in a product with a loose texture or excessive toughness. Although non-thermal sterilization techniques can minimize heat-induced damage, they are costly and may cause an off-flavor in the meat, such as an irradiation taste. Chemical sterilization, which depends on the long-term application of chemical additives, presents potential health hazards to consumers and runs counter to the current consumer inclination towards clean and natural products. In contrast, bioprotective strategies, which entail the utilization of natural microorganisms or their metabolites to inhibit or eradicate harmful microorganisms in food, offer a safer and more sustainable alternative. The incorporation of bioprotective agents can effectively impede the growth and reproduction of microorganisms through the competitive activities of the strains themselves or their antimicrobial metabolites. This not only prolongs the product’s shelf life but also guarantees its safety for consumption [3]. Additionally, bioprotective agents can enhance the sensory attributes of the meat, thereby providing consumers with an enhanced and novel eating experience [4].

Bioprotective agents predominantly encompass seaweed extracts, humic substances, protein hydrolysates, amino acids, plant extracts, and beneficial microorganisms [5]. Plants (e.g., chili peppers and herbs) are rich in various bioactive compounds (such as capsaicin, phenolics, and vanillin), which can significantly enhance microbial stability and improve antioxidant capacity [6]. However, many bioactive compounds (e.g., polyphenols, vanillin) exhibit sensitivity to light, heat, and pH variations while being susceptible to hydrolysis. Furthermore, these compounds may impart undesirable sensory characteristics (such as the pungency of capsaicin or the astringency of certain phenolic compounds), potentially restricting their utilization in food applications. At present, lactic acid bacteria, which are food-grade strains, are the bioprotective agents most extensively utilized. With the legal recognition of lactic acid bacteria as bioprotective agents, their application as bioprotective microorganisms in the food industry has gradually emerged as a research focus. Lactic acid bacteria (LAB) metabolize carbohydrates to produce organic acids (e.g., lactic acid), thereby reducing the environmental pH. Furthermore, they secrete various antimicrobial compounds, such as bacteriocins, phenolic acids, hydrogen peroxide, and exopolysaccharides. These substances disrupt the cell membrane integrity of pathogenic microorganisms, interfere with their energy metabolism, and inhibit key enzymatic activities [7]. Lopez-Arvizu et al. [8] reported that *Lactilactobacillus sakei* exhibited potent antibacterial activity against both Gram-negative and Gram-positive spoilage microorganisms. Specifically, it could effectively impede the growth and proliferation of *Escherichia coli*, *Enterococcus faecalis*, and *Micrococcus lysodeikticus*. Wang et al. [9] demonstrated that inoculating smoked chicken legs with *Pediococcus acidilactici* and *Latilactobacillus curvatus* could efficiently inhibit the growth and reproduction of common spoilage bacteria, including *Prodigibacterium*, *Weissella cetaceum*, and *Pseudomonas*. Moreover, the levels of spoilage markers such as ethanol, 1-octen-3-ol, carbon disulfide, cyclooctanol, 2,3-butanediol, and 3-methyl-1-butanol were significantly decreased. This effectively postponed the deterioration of appearance and odor, thereby extending the shelf life of smoked chicken legs. Muño et al. [10] found that lactic acid bacteria (LAB) may enhance free radical scavenging capacity and improve antioxidant activity. Parlindungan et al. [11] further found that *Lactilactobacillus sakei* exhibits broad-spectrum antibacterial activity, inhibiting *Listeria* spp. and *Staphylococcus* spp.; *Pediococcus acidilactici* demonstrates extreme acid tolerance, with a survival rate of >95% at pH 3.5, making it suitable for pickled pepper fermentation systems; *Latilactobacillus curvatus* exhibits strong antioxidative capacity, effectively suppressing *Pseudomonas* spp.

When used as probiotic cultures for the production of fermented products, LAB exhibit the efficacy of maintaining product quality. In this study, *Lactilactobacillus sakei*, *Pediococcus acidilactici*, and *Latilactobacillus curvatus* were chosen as natural biological preservatives and incorporated into pickled pepper rabbit meat and stored at 4 °C for 1, 3, 5, and 7 days. The quality characteristics (such as pH value and texture characteristics), oxidation characteristics (including TBARS value, TVB-N value content, carbonyl content, and sulfhydryl content), and microbial indicators of the products were measured. The aim was to explore the impact of *Lactilactilactobacillus sakei*, *Pediococcus acidilactici*, and *Latilactobacillus curvatus* on the quality characteristics and oxidative stability of pickled rabbit meat during refrigerated storage by regulating microbial community structure and inhibiting spoilage bacterial growth, thereby providing a scientific foundation and technical support for enhancing the processing quality of pickled pepper rabbit meat.

## 2. Materials and Methods

### 2.1. Preparation of Pickled Pepper Rabbit Meat

#### 2.1.1. Materials

Rabbit from Leshan Ha Wuye Food Co., Ltd. (Leshan, China); *Lactilactobacillus sakei* (SafePro^®^ B-SF-2), *Pediococcus acidilactici* (SafePro^®^ B-LC-20), and *Latilactobacillus curvatus* (SafePro^®^ B-LC-48), from Chr. Hansen, Germany; salt, white granulated sugar, white vinegar, pickled peppers, cooking wine, ginger, and scallions were sourced from Haolego Supermarket, Shiling Street, Chengdu, China.

#### 2.1.2. Operation Points

First, the fascia, gland and fat were removed from the fresh rabbit. The rabbit was divided and cleaned with water. The rabbit was cooked in boiling water for 15 min. Second, an appropriate amount of ginger, scallion segments and cooking wine were added into boiling water. Following this, it was cooled to room temperature and cut it into blocks of 4 cm × 4 cm × 4 cm. Meanwhile, 0.02% (*w*/*w*) bacterial strains (*Lactilactobacillus sakei*, *Pediococcus acidilactici*, and *Latilactobacillus curvatus*) were mixed with the following ingredients: water (1500 g), salt (50 g), white granulated sugar (30 g), white vinegar (200 g), and pickled peppers water (200 g). The rabbit was soaked in the solution (3:2, *w*/*w*) at 4 °C) for 24 h in the container. After this, the rabbit meat and the solution (6:1, *w*/*w*) were cooled to room temperature and vacuum packed. The production of mature fermented pickled pepper rabbit meat was completed and stored at 4 °C.

#### 2.1.3. Sampling

Four groups of pickled pepper rabbit meat were manufactured: CK (without lactic acid bacteria), LS (with *Lactilactobacillus sakei*), PA (with *Pediococcus acidilactici*) and LC (with *Latilactobacillus curvatus*). After the fermentation was completed, storage was carried out for 1, 3, 5 and 7 days at 4 °C. Three pickled pepper rabbit meat samples were taken from the control group and the fermented group for physicochemical, textural and bacterial diversity analysis. For each group of pickled pepper rabbit meat, all experiments were carried out in triplicate and the average value was taken.

### 2.2. Determination of Indexes

#### 2.2.1. pH

A 5 g sample was homogenized with 95 mL of distilled water and the pH value was detected using a pH meter (Mettler Toledo Instruments Co., Ltd., Shanghai, China).

#### 2.2.2. TBARS

The TBARS index was determined using the method of Stadnik et al. [12]. A mixed solution of 5 g of pickled pepper rabbit meat and 50 mL of trichloroacetic acid–edathamil disodium (Jinshan Chemical Reagent Co., Ltd., Chengdu, China), was shaken at 50 °C for 30 min on a constant temperature shaker (Bona Technology Co., Ltd., Chengdu, China). After this, it was cooled to room temperature and separated on double-layer filter paper. Then, 5 mL of the filtrate was mixed with 5 mL of 2-thiobarbituric acid (TBA) (0.02 mol/L) and reacted in 90 °C water for 30 min. Absorbance was measured at 532 nm using a spectrophotometer. The results were expressed as milligrams of malondialdehyde (MDA) in 1 kg of the product, as shown in the following formula:

$$TBARS[mgMDA/kg]=\frac{c\times V\times 1000}{m\times 1000}$$

#### 2.2.3. Carbonyl Content

Protein carbonyl content was evaluated from a method by Wang et al. [13] with minor modifications. A mixture of 1 g of pickled pepper rabbit meat and 1 mL of phosphate buffer (20 mmol/L) was homogenized for 2 min. An amount of 0.1 mL of 10% trichloroacetic acid (TCA) solution was added to the supernatant, and stood at room temperature for 10 min. Then, the mixtures were centrifuged (5000× *g*, 10 min, 4 °C), and the supernatant was collected. After this, 160ul of the supernatant from the control tube and 120 μL of 2,4-dinitrophenylhydrazine (DNPH) solution (containing 2 mol/L HCl) were mixed. We took 160 μL of the supernatant from the test tube and added 120 μL of HCl solution (2 mol/L), and reacted at 37 °C in the dark for 1 h, shaking once every 10 min. After the reaction, we added 150 ul of trichloroacetic acid (TCA) and the mixture was left to stand for 5 min. Then, the mixtures were centrifuged (12,000× *g*, 15 min, 4 °C), and the supernatant was discarded. The filtrate was washed with 1mL of ethanol–ethyl acetate (1:1 [*v*/*v*]) three times. Afterwards, the resulting pellet was dissolved in 200 ul of phosphate buffer (20 mmol/L) and incubated at 37 °C for 15 min. Finally, the mixtures were centrifuged (12,000× *g*, 15 min, 4 °C), and 200 μL of the supernatant was taken to measure the absorbance at 370 nm. The carbonyl content was calculated using the following formula:

$$Carbonyl\;Content/(\mu mol/g)=\frac{A_{Measurement\;tube}-A_{Control\;tube}}{16\times W}$$

#### 2.2.4. Sulfhydryl Content

The sulfhydryl content index was determined using the method of Chen et al. [14]. We mixed 0.1 g of the pickled pepper rabbit meat sample and 1 mL of methanol solution (38%). The mixtures were centrifuged (8000× *g*, 10 min) at room temperature, and the supernatant was collected. Then, 40 μL of supernatant and 150 μL of HCl solution (2 mol/L) were diluted in the test tube and control tube, respectively. We added 10 μL of paraformaldehyde solution to the control tube, and added 10 μL of pure water to the test tube. These stood at room temperature for 10 min and the absorbance was measured at 412 nm. The sulfhydryl (SH) content was calculated using the following formula:

$$Sulfhydryl\;(SH)\;content/(\mu mol/g)=\frac{x}{W}$$

#### 2.2.5. Total Volatile Basic Nitrogen (TVB-N)

TVB-N was evaluated from a method by Cha et al. [15]. An amount of 10 g pickled pepper rabbit meat underwent homogenization in 100 mL distilled water (30 min), followed by filtration. A 10 mL aliquot of the filtrate was combined with 10 mL MgO solution (10 g/L) and distilled via a KDN—102 C nitrogen analyzer (Fiber Inspection Instrument Co., Ltd., Shanghai, China). Volatile bases were captured in 20 mL boric acid solution (20 g/L) containing a dual-indicator system (1.0 g/L methylene blue and methyl red in 95% ethanol). Titration was conducted with 0.01 mol/L HCl quantified nitrogen content, with a blank (10 mL water substituted for sample) correcting background interference. TVB-N values were calculated as mg N/100 g meat, with triplicate measurements ensuring reproducibility.

#### 2.2.6. Texture Profile Analysis (TPA)

The Texture Profile Analysis index was determined using the method of Luo et al. [16]. All pickled pepper rabbit meat was cut into cubic pieces of 1 cm × 1 cm × 1 cm before analysis. Texture Profile Analysis (TPA) was performed on samples using a texture analyzer (TA-XT Plus, STab Ltd., Godalming, UK) equipped with a P/36R cylindrical probe. Pre-test speed, test speed and post-test speed were all 2 mm/s and the compression ratio was set to 50%. The measurement was cycled twice, and a trigger force of 8 g was used to evaluate the texture characteristics of the pickled pepper rabbit meat samples.

#### 2.2.7. Microbiological Analysis

A total of 10.0 g of pickled pepper rabbit meat was homogenized with 90 mL of sterile saline for 5 min. Continuously dilution with sterile saline was performed to obtain a series of 10-fold dilutions, and 100 μL of the appropriate dilutions were spread on the corresponding plate count agar and MRS agar dishes for incubation at 30 ± 1 °C for 48 h.

#### 2.2.8. High-Throughput Sequencing

The high-throughput sequencing was determined using the method of Casaburi et al. [17]. The sample DNA was purified using the Zymo Research BIOMICS DNA Microprep Kit (Cat# D4301) (Orange County, CA, USA) to extract genomic DNA (gDNA). The integrity of the gDNA was assessed via 0.8% agarose gel electrophoresis, followed by nucleic acid concentration measurement using the Tecan F200 (Zurich, Switzerland) with the PicoGreen dye method. The 16S rRNA gene was amplified using the primers 338F (5′-ACTCCTACGGGAGGCAGCAG-3′) and 806R (5′-GGACTACHVGGGTWTCTAAT-3′). The PCR protocol was as follows: initial denaturation at 94 °C for 1 min (1 cycle); denaturation at 94 °C for 20 s; annealing at 54 °C for 30 s and extension at 72 °C for 30 s (25–30 cycles); final extension at 72 °C for 5 min (1 cycle); and holding at 4 °C. Each sample was subjected to three technical replicates of PCR, and the PCR products from the linear amplification phase were pooled in equal amounts for subsequent library preparation. The PCR products were mixed with 6× loading buffer and electrophoresed on a 2% agarose gel to verify the target fragments. Qualified samples were excised for gel extraction using the Zymoclean Gel Recovery Kit (D4008) (Orange County, CA, USA). The purified products were quantified using the Qubit^®^ 2.0 Fluorometer (Thermo Scientific, Waltham, MA, USA) and pooled in equimolar amounts. Library construction was performed using the NEBNext Ultra II DNA Library Prep Kit for Illumina (NEB#E7645L) from New England BioLabs (Worcester, MA, USA). High-throughput sequencing was conducted using the PE250 mode on the Illumina NovaSeq 6000 platform with the NovaSeq 6000 SP Reagent Kit V1.5 (Illumina San Diego, CA, USA).

Alpha diversity indices (including the Shannon index, Simpson index, and Chao1 index) were employed to evaluate the diversity and richness of microbial communities within the samples [18]. To mitigate biases arising from uneven sequencing depth, all samples were rarefied to an identical sequencing depth prior to operational taxonomic unit (OTU) clustering. The Shannon and Simpson indices were calculated based on the normalized OTU abundance table, reflecting species diversity, while the Chao1 index was used to estimate community richness.

#### 2.2.9. Statistical Analysis

The data obtained from the experiment were processed using IBM SPSS Statistics (29.0.1.0). One-way analysis of variance (ANOVA) was used to determine the significance of the differences, with *p* < 0.05 as the significance level. The data were visualized using Origin 2021 and are expressed as the mean ± standard deviation (SD) of three replicate determinations.

## 3. Results

### 3.1. pH

**Figure 1:**
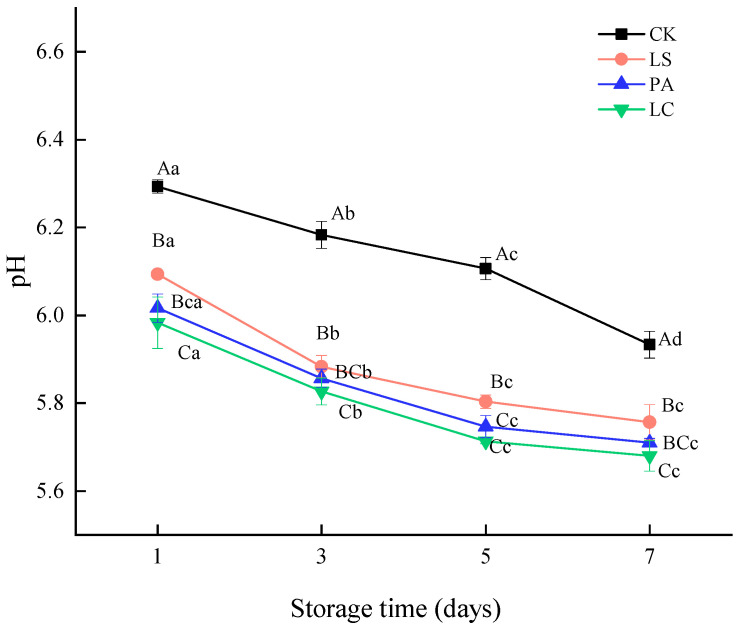
Effect of different lactic acid bacteria bioprotectants on the pH of pickled pepper rabbit meat. Different uppercase letters indicate significant differences (*p* < 0.05) between groups at the same storage time, while different lowercase letters indicate significant differences (*p* < 0.05) within the same group across different storage times. Abbreviations: CK, control; LS, *Lactilactilactobacillus sakei*; PA, *Pediococcus acidilactici*; LC, *Lactilactilactobacillus curvatus*. This notation applies consistently to Figure 1, Figure 2, Figure 3, Figure 4, Figure 5, Figure 6 and Figure 7.

The changes in pH of rabbit meat samples during storage are shown in Figure 1. With the extension of storage time, the pH of the control, LS, PA, and LA treatments decreased significantly from 6.29, 6.07, 6.01, 5.98 (1 d) to 5.93, 5.76, 5.71, and 5.68 (7 d) (*p* < 0.05), respectively, which were 5.72%, 5.21%, 5.10%, and 5.07% (7 d) lower than those after 1 day (*p* < 0.05). The results indicate that the addition of lactic acid bacteria effectively delays the decline of pH of pickled pepper rabbit meat during storage. Furthermore, at the same storage period, the pH of the LS, PA, and LC treatments was significantly lower than that of the control (*p* < 0.05). Especially, the pH, respectively, was 3.50%, 4.39%, and 4.93% lower than the control after 1 day. The results indicate that the addition of these three lactic acid bacteria effectively prevents the increase of pH of pickled pepper rabbit meat in the early stages of storage.

### 3.2. TBARS

**Figure 2:**
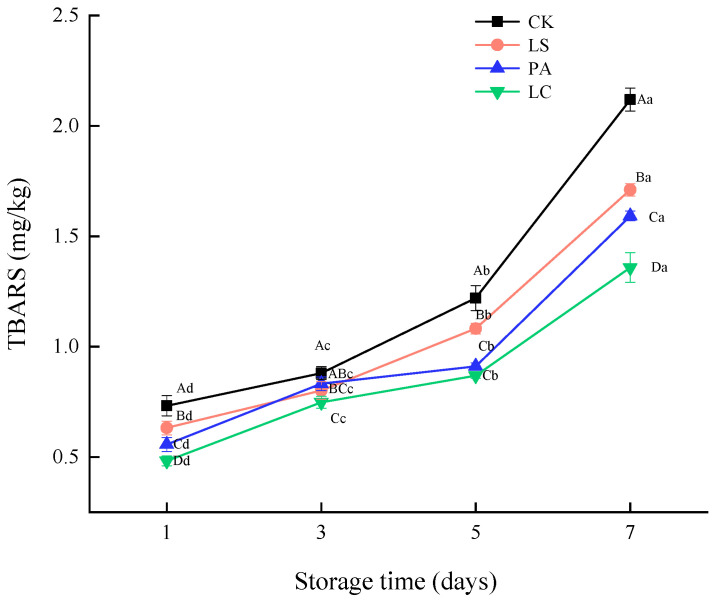
Effect of different lactic acid bacteria bioprotectants on the TBARS in pickled pepper rabbit meat.

Figure 2 shows the changes in the thiobarbituric acid reactive substances (TBARS) values of pickled pepper rabbit meat during storage. As the storage time progresses, the TBARS values of pickled pepper rabbit meat in the control group, LS group, PA group, and LC group increase significantly. Specifically, they increase from 0.73, 0.63, 0.56, and 0.48 on day 1 to 2.11, 1.71, 1.59, and 1.36 on day 7 (*p* < 0.05), representing increases of 65.43%, 63.04%, 65.00%, and 64.44%, respectively. The results indicated that the lipid oxidation of samples in each group was exacerbated during the storage period. However, the experimental group inoculated with lactic acid bacteria exhibited a significantly lower level of lipid oxidation compared to the control group (*p* < 0.05). Notably, the LC group (*Latilactobacillus curvatus*) demonstrated the lowest oxidation level. In addition, within the same storage period, the TBARS values of the LS, PA, and LC groups were significantly lower than the control group (*p* < 0.05). Specifically, after 7 days of storage, the TBARS values of treatment groups were, respectively, 19.26%, 24.86%, and 35.89% lower than the control group (*p* < 0.05). The results demonstrated that the three types of lactic acid bacteria could effectively inhibit lipid oxidation. Among them, *Latilactobacillus curvatus* showed the most remarkable inhibitory effect on lipid oxidation (*p* < 0.05).

### 3.3. Carbonyl Content

**Figure 3:**
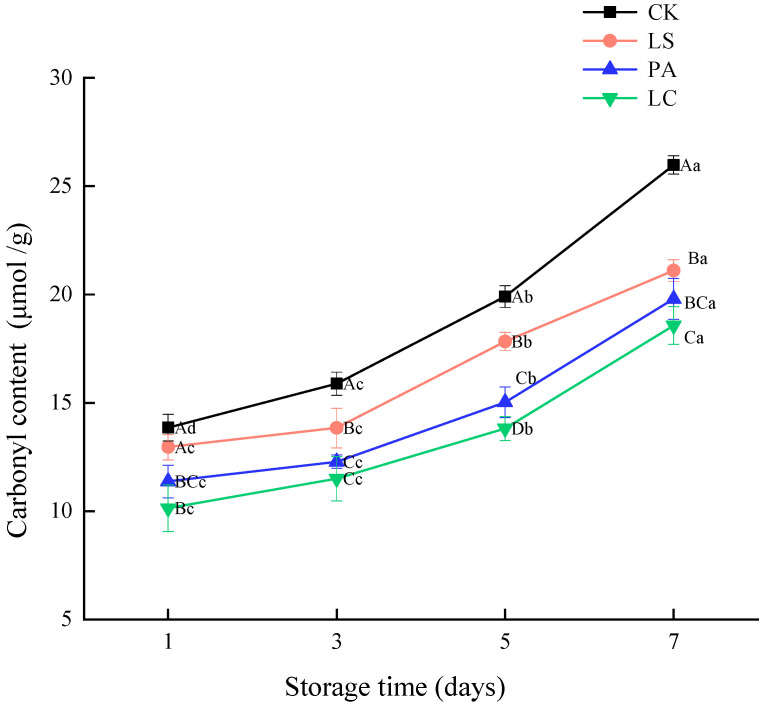
Effect of different lactic acid bacteria bioprotectants on the carbonyl content in pickled pepper rabbit meat.

Figure 3 illustrates the changes in the carbonyl content of pickled pepper rabbit meat during storage. As the storage time progresses, the carbonyl content of the pickled pepper rabbit meat in the control group, LS group, PA group, and LC group increases significantly. The carbonyl content of the pickled pepper rabbit meat gradually increased from 13.86, 12.96, 11.37, and 10.14 on day 1 to 25.98, 21.11, 19.80, and 18.57, respectively, on day 7 (*p* < 0.05), showing increases of 46.66%, 38.61%, 42.55%, and 45.39%, respectively. The results indicated that the carbonyl content of each group increased significantly as the storage time extended (*p* < 0.05). In addition, the carbonyl content of the LS, PA and LC groups was significantly (*p* < 0.05) lower than that of the control group. Notably, the carbonyl content in the LS, PA and LC groups was, respectively, 18.75%, 23.79%, and 28.53% lower than that of the control group on day 7. This result indicates that the addition of lactic acid bacteria could effectively inhibit protein oxidation. Notably, the inhibitory effects of the PA and LC groups were particularly prominent (*p* < 0.05).

### 3.4. Sulfhydryl Content

**Figure 4:**
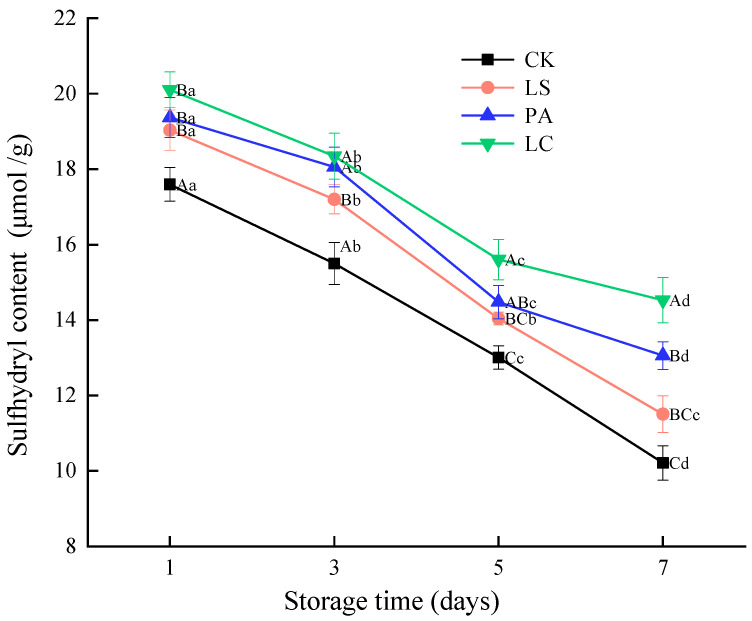
Effect of different lactic acid bacteria bioprotectants on the sulfhydryl content in pickled pepper rabbit meat.

Figure 4 illustrates the changes in the sulfhydryl content of pickled pepper rabbit meat during storage. As the storage time progresses, the sulfhydryl content of the pickled pepper rabbit meat decreased significantly from 17.75, 19.03, 19.37, and 20.10 on day 1 to 10.21, 11.51, 13.06, and 14.53, respectively, on day 7 (*p* < 0.05), a decrease of 42.45%, 39.54%, 32.58%, and 27.72%, respectively. The results revealed that the sulfhydryl content in each group decreased significantly over storage time (*p* < 0.05), while the degree of protein oxidation increased significantly. However, a significant difference was observed in the decline extent. Specifically, the decline rate of the lactic acid bacteria group was significantly lower than that of the control group. This indicates that lactic acid bacteria can effectively slow down the loss of sulfhydryl groups, protect the protein structure, and exhibit excellent antioxidant properties. It has been demonstrated that the sulfhydryl content of each group decreased significantly during storage (*p* < 0.05), and the degree of protein oxidation increased remarkably over time. However, the extent of decline varied significantly between groups. In addition, the sulfhydryl content of the CK, LS and PA groups was significantly lower than that of the LC group (*p* < 0.05). Notably, the sulfhydryl content in CK, LS and PA groups was, respectively, 29.71%, 20.81%, and 10.12% lower than that of the LC group on day 7. This implies that the LC group was found to have the most potent inhibition of protein oxidation.

### 3.5. TVB-N

**Figure 5:**
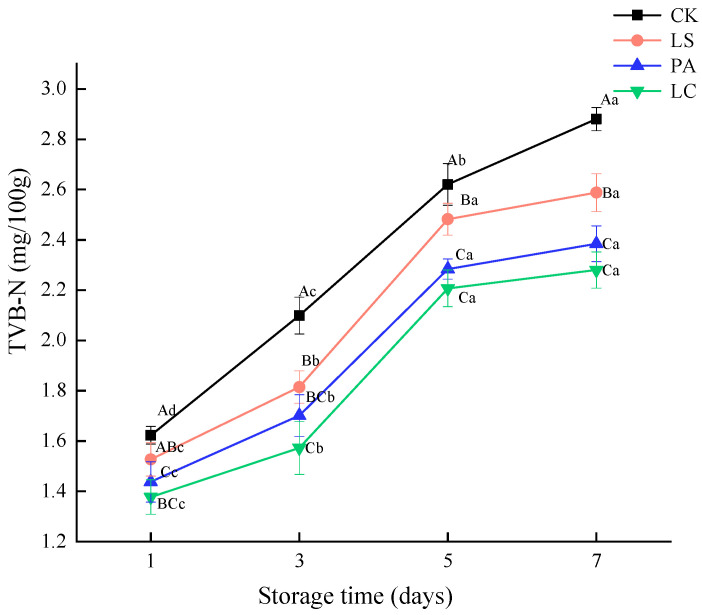
Effect of different lactic acid bacteria bioprotectants on the TVB-N in pickled pepper rabbit meat.

Figure 5 depicts the effects of different Lactobacillus-based bioprotective cultures on TVB-N values in pickled pepper rabbit meat. As the storage time progresses, TVB-N content in all groups (CK, LS, PA, and LC), significantly increased from 1.62, 1.53, 1.44, and 1.38 on day 1 to 2.88, 2.59, 2.38, and 2.28, respectively, on day 7 (*p* < 0.05), an increase of 43.66%, 41.02%, 39.71%, and 39.61%, respectively. The results demonstrate that TVB-N content increased significantly with prolonged storage time. On day 7, TVB-N values in the LS, PA, and LC groups were 10.14%, 17.23%, and 20.84% lower than those in the CK group, respectively (*p* < 0.05). These results indicate that rabbit meat without lactic acid bacteria treatment is more susceptible to protein degradation and spoilage during storage. The LC group showed the most significant inhibitory effect on protein oxidation.

### 3.6. Texture Profile Analysis (TPA)

**Figure 6:**
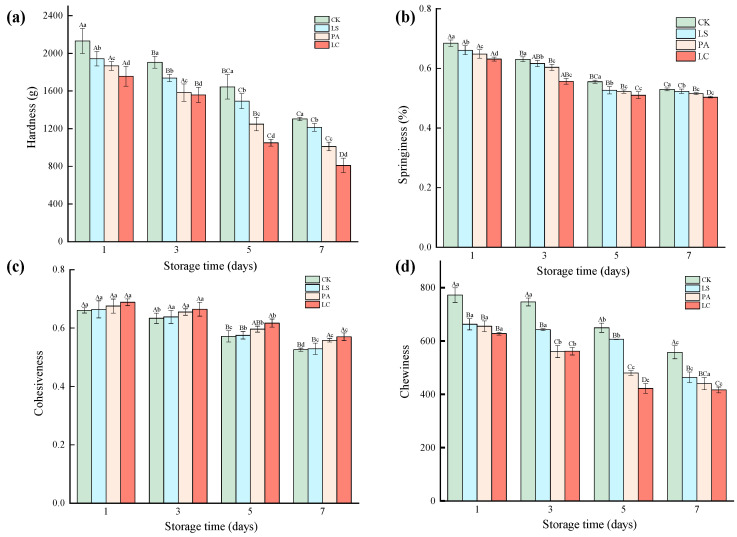
Effect of different lactic acid bacteria bioprotectants on the texture characteristics of pickled pepper rabbit meat. (**a**) Hardness; (**b**) spring; (**c**) cohesiveness; (**d**) chewiness.

As shown in Figure 6, the addition of lactic acid bacteria bioprotectants significantly affected (*p* < 0.05) the texture characteristics (including hardness, springiness, resilience, cohesiveness, and chewiness) of pepper-pickled rabbit meat. In this study, the hardness of pickled pepper rabbit meat decreased significantly during storage. Compared with day 1, the elasticity of hardness reductions in the CK, LS, PA, and LC groups were 38.87%, 37.46%, 45.88%, and 53.85% on day 7 (*p* < 0.05). Throughout storage, the CK group maintained significantly higher hardness than the treated groups, with values of 6.78%, 22.48%, and 37.82% higher than the LS, PA, and LC groups on day 7, respectively, with the LC group showing the lowest hardness. On day 7, the elasticity of lactic acid bacteria-treated rabbit meat was significantly lower than that of the CK group, with the LS, PA, and LC groups showing 1.23%, 2.64%, and 4.91% reductions, respectively. Compared with the CK group, the cohesiveness of lactic acid bacteria-inoculated rabbit meat increased significantly during storage. On day 7, the cohesiveness of the LS, PA, and LC groups was 0.62%, 5.75%, and 7.78% higher than that of the CK group, respectively (*p* < 0.05). Furthermore, the chewiness of lactic acid bacteria-inoculated rabbit meat was significantly lower than that of the CK group. On day 7, the LS, PA, and LC groups showed 16.86%, 21.13%, and 25.32% reductions in chewiness compared to the control, respectively (*p* < 0.05).

### 3.7. Microbiological Analysis

**Figure 7:**
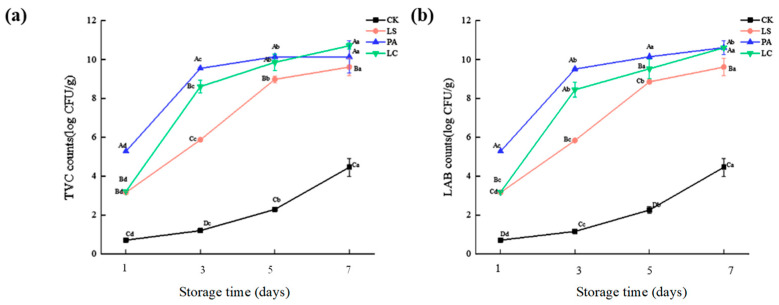
Effect of different lactic acid bacteria bioprotectants on the texture characteristics of pickled pepper rabbit meat. (**a**) LAB counts; (**b**) TVC counts.

Figure 7 illustrates the dynamics of TVC counts and LAB counts in pickled pepper rabbit meat during storage. On day 1, LAB counts in treated groups (LS: 3.17; PA: 5.28; LC: 4.46 log CFU/g) significantly exceeded control levels (0.71 log CFU/g; *p* < 0.05). By day 7, all groups exhibited significant increases (*p* < 0.05) in LAB counts, with increments of 6.45, 4.25, and 6.15 log units for LS, PA, and LC treatments, respectively, versus 3.75 log units in the control. The results demonstrated that inoculation with diverse lactic acid bacteria (LAB) bioprotective cultures significantly influenced the microbial dynamics of pickled pepper rabbit meat during both initial colonization and storage phases (*p* < 0.05). Specifically, the LC group exhibited the highest LAB counts, exceeding those of the CK, LS, and PA groups by 6.15, 0.99, and 1.08 log units, respectively. This indicates that *Latilactobacillus curvatus* possessed the most significant value-enhancing effects on LAB proliferation.

Since the inoculation of lactic acid bacteria significantly affected TVC, the overall trend of TVC was similar to that of LAB counts. The initial TVC counts in the LS, PA, and LC groups were 3.17 log CFU/g, 5.28 log CFU/g, and 3.22 log CFU/g, respectively, which were significantly higher than the TVC counts of the CK group (0.71 log CFU/g). After 7 days of storage, a consistent and substantial increase in TVC was observed across all groups. Specifically, the TVC counts increased by 6.45, 5.19, and 7.49 log units in the LS, PA, and LC treatments, respectively, and by 3.75 log units in the CK group compared to day 1. The increase was most pronounced in the LC group (*p* < 0.05). Furthermore, the TVC counts of the treatment groups inoculated with lactic acid bacteria bioprotectants were significantly higher than those of the CK group on day 7. The TVC counts in the LS, PA, and LC groups were 4.54, 6.01, and 6.25 log units higher than that of the CK group, respectively.

### 3.8. Microbial Diversity

**Figure 8:**
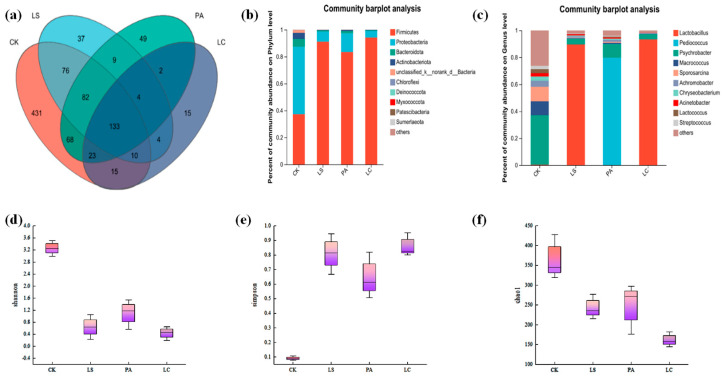
Effect of different lactic acid bacteria bioprotectants on the microbial diversity of pickled pepper rabbit meat. (**a**) OTU-level Venn diagram; (**b**) bacterial phylum-level relative abundance; (**c**) bacterial genus-level relative abundance; (**d**) Shannon index; (**e**) Simpson index; (**f**) Chao1 index.

As shown in Figure 8a, 133 OTUs were shared across all four treatment groups (CK, LS, PA, and LC) on day 7, representing the core bacterial community. The total OTU counts for each group were 838 (CK), 355 (LS), 370 (PA), and 206 (LC), respectively. Among these, the CK group exhibited the highest number of unique OTUs (431), accounting for 91.53%, 88.63%, and 96.52% of the unique OTUs in the LS, PA, and LC groups, respectively. The results demonstrate that the LC group had the lowest OTU richness. As shown in Figure 8b, the phylum-level bacterial composition of pickled pepper rabbit meat varied significantly among different treatment groups after 7 days of storage. Firmicutes emerged as the dominant phylum in all groups inoculated with LAB bioprotective cultures, accounting for 91.16%, 83.44%, and 94.25% of the relative abundance in LS, PA, and LC groups, respectively.

Notably, the relative abundance of Firmicutes was significantly higher in bioprotectant-treated groups compared to the CK group, suggesting successful proliferation of fermentation microbiota in the pickled pepper rabbit meat. Particularly, the LC group showed the most pronounced dominance (94.25% relative abundance), indicating the most substantial growth of LC strain, which aligns perfectly with the highest LAB counts observed in Section 3.7. In contrast, *Proteobacteria* dominated in the CK group, representing 50.04% of the total phylum-level abundance.

The genus-level bacterial composition of pickled pepper rabbit meat across different treatments is shown in Figure 8c, demonstrating that LAB bioprotective cultures significantly altered the microbial community structure. In the CK group, *Psychrobacter* dominated (36.60%), while the LAB-treated groups showed distinct dominant genera: *Lactobacillus* (89.66%) in LS, *Pediococcus* (79.70%) in PA, and *Lactobacillus* (93.50%) in LC. Notably, *Psychrobacter* is a predominant spoilage organism in refrigerated foods, indicating increased spoilage risk during storage of the control samples. Importantly, LAB inoculation markedly reduced *Psychrobacter* abundance by 32.29%, 26.55%, and 32.95% in the LS, PA, and LC groups, respectively, compared to CK, demonstrating LAB’s ability to inhibit spoilage bacteria growth through acidification [19].

As illustrated in Figure 8d, the CK group exhibited significantly higher Shannon indices (*p* < 0.05) compared to LAB-treated groups, indicating both greater species evenness and enhanced ecological diversity in control samples. In contrast, LAB inoculation induced pronounced species dominance, resulting in decreased α-diversity. This phenomenon was further corroborated by Simpson index analysis (Figure 8e), where LAB-treated groups showed significantly higher values (*p* < 0.05), confirming the establishment of simplified community structures dominated by single strains. The Chao1 index further corroborated these results. Specifically, the CK group demonstrated the highest level of community diversity. The LS and PA groups exhibited comparable diversity levels, while the LC group manifested the lowest diversity. The results showed complete concordance with both the OTU clustering patterns and phylum-level distribution profiles, collectively indicating that the LC group developed the most simplified microbial ecosystem. This phenomenon revealed that *Latilactobacillus curvatus* dominated the LC group’s microbiota, effectively establishing a near-monoculture state.

## 4. Discussion

The pH is one of the most important indicators in assessing the quality of meat, which can affect the flavor, texture, and safety of products [20]. The significant decrease in pH of pickled pepper rabbit meat during the storage may be due to the production of acidic substances from gradual proliferation and fermentation metabolism of natural microbiota or spoilage bacteria [21]. Mejri et al. [22] found that during fermentation, the high activity of fermentation enzymes in meat led to the breakdown of glycogen, producing acidic substances such as lactic acid and phosphoric acid, which caused the decrease of pH. However, the lower decrease rate of pH of treatments may be due to the rapid dominance of lactic acid bacteria in the early stage of fermentation, inhibiting the growth of other harmful microorganisms, thus stabilizing the microbial community structure in the later stages of storage and preventing significant fluctuations of pH [23]. The addition of *Lactilactobacillus sakei*, *Pediococcus acidilactici*, and *Latilactobacillus curvatus* effectively prevents the increase of pH in pickled pepper rabbit meat in the early stages of storage. This may be because the three lactic acid bacteria with high metabolic activity could produce sufficient lactic acid in a short period to prevent the increase of pH. Shang et al. [24] found that the rapidly increasing lactic acid bacteria in acidic meat could take advantage of the sugars in the meat for metabolism, generating a large amount of lactic acid, which significantly reduced the pH of the system. Narasimulu et al. [25] demonstrated that low pH can inhibit the growth and proliferation of pathogens and spoilage bacteria, enhancing the safety of products.

Lipolysis facilitates lipid oxidation, which leads to the deterioration of fresh meat quality [26]. Malondialdehyde is the main by-product of lipid peroxidation. In meat products, a higher malondialdehyde value indicates a more severe degree of lipid oxidation [27]. The experimental group inoculated with lactic acid bacteria exhibited a significantly lower level of lipid oxidation compared to the control group (*p* < 0.05). Moreover, the LC group (*Latilactobacillus curvatus*) demonstrated the lowest oxidation level. This phenomenon could be attributed to the fact that lactic acid bacteria inhibit the activity of lipolytic enzymes, thereby reducing the generation of free fatty acids. Additionally, lactic acid bacteria produce certain substances through their metabolic activities that contribute to the stabilization of lipids and enhance antioxidant capacity. Gao et al. [28] found that when inoculated with lactic acid bacteria, the proportion of monounsaturated fatty acid in fermented mutton sausage increased significantly, and fat peroxidation was effectively inhibited. During the entire storage period, the TBARS value of the fermented mutton sausage supplemented with lactic acid bacteria was significantly lower than that of the control group (*p* < 0.05). Fıçıcılar et al. [29] further discovered that the addition of lactic acid bacteria had the potential to boost the antioxidant activity and decrease the generation of reactive oxygen species (ROS). Moreover, the TBARS value of hot-smoked rainbow trout was significantly lower than that of the control group during the storage period (*p* < 0.05).

The results of this study demonstrate that *Lactilactobacillus sakei*, *Pediococcus acidilactici*, and *Latilactobacillus curvatus* could effectively inhibit lipid oxidation. Among these, *Latilactobacillus curvatus* showed the most remarkable inhibitory effect on lipid oxidation (*p* < 0.05). This phenomenon may be ascribed to the disparities in metabolic capacity, acid production capacity and antioxidant metabolite synthesis capacity of different lactic acid bacteria, and *Latilactobacillus curvatus* may have stronger antioxidant activity. Zhang et al. [30] found that *Latilactobacillus curvatus* demonstrated remarkable antioxidant capacity. Furthermore, the augmentation of its DPPH scavenging activity and reducing power was directly correlated with its capacity to impede lipid oxidation and lower the TBARS value. *Latilactobacillus curvatus* can effectively inhibit the chain reaction of fatty acid oxidation by scavenging free radicals and reducing oxides. Moreover, it effectively reduced the TBARS value of pickled pepper rabbit meat, which was consistent with the research findings of Chen et al. [31].

Carbonylation is generally considered an irreversible non-enzymatic chemical modification reaction in protein oxidation [32]. Carbonylation serves as a primary marker of protein oxidation, effectively characterizing the degree of oxidative modification in proteins. Specifically, a higher carbonyl content implies a greater extent of protein oxidation [33]. The carbonyl content of each group increased significantly as the storage time extended (*p* < 0.05), which could be attributed to the fact that meat intrinsically contains various oxygen-promoting factors, including unsaturated lipids, heme pigments, metal ions, and endogenous oxidases, which have triggered the oxidation chain reaction in the initial stage of fermentation [34]. During the fermentation process, the unsaturated lipids in pickled pepper rabbit meat are oxidized, generating a series of oxidation by-products, such as alkoxy, peroxy radicals, and aldehydes. These by-products possess strong electrophilicity and reactivity, and can significantly expedite the oxidation process of proteins. Al-Sahlany et al. [35] found that lactic acid bacteria promote the release of polyphenols by fermenting to produce lactic acid and β-glucosidase, thereby enhancing antioxidant capacity. Wang et al. [13] found that the lipid oxidation of rabbit meat could significantly increase the denaturation rate of myoglobin and myofibrillar protein and promote protein carbonylation. The addition of lactic acid bacteria could effectively inhibit protein oxidation, and the PA and LC groups maintained the carbonyl content at a relatively low level. Chen et al. [14] discovered that adding the *Latilactobacillus curvatus* to fermented meat products produced antioxidants and decreased the carbonyl content. This implies that *Latilactobacillus curvatus* inhibits protein oxidation, slows down the hydrolysis of sarcoplasmic protein and myofibrillar protein, and reduces the generation of carbonyl compounds during the fermentation process. Li et al. [36] discovered that the inoculation of *Pediococcus acidilactici* could enhance the activity of cathepsin B and cathepsin L. Moreover, it could decelerate the oxidation process of myofibrillar protein and sarcoplasmic protein during the fermentation stage. Furthermore, Berardo et al. [37] indicated that a lower pH value can effectively impede the oxidation of sample proteins during the fermentation process, which is in accordance with the result presented in Section 3.1. This phenomenon might be attributed to the rapid multiplication of lactic acid bacteria within a short period, which utilized the sugars in the meat for metabolic processes, generating large amounts of lactic acid with antioxidant activity. This consequently inhibited protein oxidation and reduced the formation of carbonyl compounds [38].

The sulfhydryl groups (-SH) in the sulfur-containing amino acids of proteins are unstable and highly vulnerable to oxidation-induced destruction, which results in a decrease in their content. Consequently, the extent of sulfhydryl loss serves as a reliable indicator of protein oxidation severity. Specifically, the lower total sulfhydryl content indicates the more severe degree of protein oxidation [36]. The lactic acid bacteria group exhibited a markedly slower rate of sulfhydryl loss compared to the control group, indicating that lactic acid bacteria can effectively exert potent antioxidant effects, protect protein structure, and delay sulfhydryl depletion. Stadnik et al. [12] demonstrated that, compared to the control group, dry-cured meat inoculated with lactic acid bacteria exhibited lower protein surface hydrophobicity and higher sulfhydryl compound content, indicating that lactic acid bacteria inhibit protein oxidation in meat samples. This can be attributed to the presence of antioxidant compounds in lactic acid bacteria, such as superoxide dismutase, catalase, glutathione peroxidase, nicotinamide adenine dinucleotide oxidase, peroxidase, and glutathione, which scavenge or neutralize reactive oxygen species and free radicals [39]. This implies that the antioxidant activity of pickled pepper rabbit meat inoculated with lactic acid bacteria is enhanced, thereby reducing free radical attack on protein sulfhydryl groups and delaying lipid peroxidation chain reactions. The LC group was found to have the most potent inhibition of protein oxidation, which may be attributed to the ability of *Latilactobacillus curvatus* to efficiently decompose sarcoplasmic proteins and produce diverse small-molecular-weight antioxidant peptides [40]. Chen et al. [14] demonstrated that inoculating *Latilactobacillus curvatus* into Harbin dry sausages significantly reduced sulfhydryl group loss and suppressed protein oxidation. This effect could be linked to the substantial lactic acid production by *Latilactobacillus curvatus*, which enhances antioxidant activity. Casaburi et al. [17] further discovered that *Latilactobacillus curvatus* exhibited excellent acidification capabilities, was abundant in superoxide dismutase, and possessed highly active aminopeptidase. This bacterium effectively hydrolyzed sarcoplasmic proteins and inhibited myofibrillar protein oxidation, thereby delaying overall protein oxidation.

Total volatile basic nitrogen (TVB-N) content is a crucial indicator for evaluating meat freshness and an important criterion for assessing spoilage [41]. TVB-N content increased significantly with prolonged storage time. This phenomenon may be attributed to the degradation of meat proteins by microorganisms and endogenous enzymes during storage, which releases nitrogenous compounds and increases TVB-N values [42]. Rabbit meat without lactic acid bacteria treatment is more susceptible to protein degradation and spoilage during storage. The LC group showed the most significant inhibitory effect on protein oxidation. Li et al. [43] further found that lactic acid bacteria inoculation suppressed protein decomposition in shrimp by reducing nitrogenous substance breakdown, thereby inhibiting TVB-N increases. Similarly, Sun et al. [44] demonstrated that *Latilactobacillus curvatus* in dry sausages restrained TVB-N elevation, likely by inhibiting N-nitrosamine accumulation during fermentation.

Throughout storage, the CK group maintained significantly higher hardness than the treated groups, which may result from lactic acid bacteria secreting extracellular polysaccharides (EPS), since these gelling compounds fill intermuscular gaps. Hilbig et al. [45] reported that *Lactilactobacillus sakei* and *Latilactobacillus curvatus* produced ex-opolysaccharides, which significantly reduced sausage hardness (*p* < 0.05). Notably, sausages inoculated with *Latilactobacillus curvatus* exhibited the lowest hardness values. The elasticity of lactic acid bacteria-treated rabbit meat was significantly lower than that of the CK group, which may be attributed to extracellular proteases actively secreted by lactic acid bacteria, which degrade proteins and reduce elasticity. Lou et al. [16] further demonstrated that such proteases promote proteolysis, leading to reduced hardness and elasticity in sardine strips. The cohesiveness of lactic acid bacteria-inoculated rabbit meat increased significantly during storage, which may be attributed to extracellular polysaccharides produced by lactic acid bacteria. These polysaccharides fill intermyofibrillar spaces, enhance protein–protein interactions, and form a three-dimensional network structure, thereby improving cohesiveness [46]. Riebroy et al. [47] further demonstrated that pH reduction alters fibrin structure and induces acid gelation, consequently affecting cohesiveness. The chewiness of lactic acid bacteria-inoculated rabbit meat was significantly lower than that of the CK group. This observation aligns with Slima et al. [48], who reported significantly decreased chewiness in lactic acid bacteria-treated meat products. Similarly, Trabelsi et al. [49] further demonstrated that lactic acid bacteria inoculation notably reduced both elasticity and chewiness in beef.

Lactic acid bacteria (LAB) and total viable counts (TVC) were analyzed in pepper-pickled rabbit meat during storage (Figure 7a,b). LAB are core microorganisms in meat fermentation, producing various bioactive metabolites that enhance food preservation and quality [50]. The inoculation with diverse lactic acid bacteria (LAB) bioprotective cultures significantly influenced the microbial dynamics of pickled pepper rabbit meat during both initial colonization and storage phases. This trend was consistent with observations in Lactobacillus-inoculated beef [49]. *Latilactobacillus curvatus* possessed the most significant value-enhancing effects on LAB proliferation. Castellano et al. [51] reported that *Latilactobacillus curvatus* effectively promoted LAB growth in vacuum-packed fresh beef, with counts increasing from 6.10 to 8.40 log CFU/g during storage. Furthermore, *Latilactobacillus curvatus* became the dominant microbial population and extended product shelf life without compromising microbiological safety.

Total viable count (TVC) is widely employed to assess the microbiological quality and shelf life of food. However, when lactic acid bacteria are introduced into meat as a biological preservative, they can selectively impede the growth of endogenous spoilage bacteria. Consequently, the conventional TVC method is no longer appropriate for evaluating the spoilage status of meat samples, and a comprehensive evaluation of bacterial diversity is essential [52]. The TVC counts of the treatment groups inoculated with lactic acid bacteria bioprotectants were significantly higher than those of the CK group on day 7. A comparable trend was also observed in smoked chicken legs inoculated with lactic acid bacteria bioprotectants (LS, LC). After 28 days of storage, the TVC in these groups increased significantly by 1.54 and 1.64 log units and was significantly higher than that of the control group (*p* < 0.05) [9].

The level of the bacterial operational taxonomic unit (OTU) serves as an indicator of microbial community diversity and composition. A higher OTU value indicates a greater number of distinct microbial species in the sequencing samples. The LC group had the lowest OTU richness. The inoculation of LAB bioprotectants significantly reduced bacterial diversity in pickled pepper rabbit meat. This phenomenon may be attributed to the dominance of LAB likely suppressed the growth of other microbiota, leading to a simplified community structure, consistent with findings reported by Shao et al. [53]. Firmicutes emerged as the dominant phylum in all groups inoculated with LAB bioprotective cultures. This dominance might be attributed to the fact that common bacterial genera in fermented meat products (Weissella, *Lactobacillus*, *Staphylococcus*, and *Lactococcus*) all belong to the Firmicutes phylum [53]. *Proteobacteria* dominated in the CK group, and it likely served as the primary microbial population in the meat samples. The effective suppression of *Proteobacteria* growth by LAB bioprotectants is consistent with findings by Liu et al. [54], who reported that LAB can produce various antimicrobial compounds (e.g., lactic acid and bacteriocins) to inhibit pathogens and spoilage microorganisms, thereby reducing the proportion of *Proteobacteria*.

The Shannon and Simpson indices were employed to evaluate microbial community diversity, simultaneously characterizing both species abundance and distribution uniformity within samples. The elevated Shannon values corresponded with reduced microbial diversity, while the Simpson index demonstrated an inverse relationship. The Chao1 index was used to assess bacterial species richness [55]. *Latilactobacillus curvatus* dominated the LC group’s microbiota, effectively establishing a near-monoculture state. Gajendran et al. [56] reported that lactic acid bacteria in biopreservation utilizing harmless microbes or their byproducts enhances food safety and longevity. LAB possess various inhibitory mechanisms that disrupt the formation of pathogenic microorganisms. Lactic acid bacteria secrete a variety of secondary metabolites with antimicrobial properties to prevent competing organisms from occupying the same ecological niche. Janßen et al. [57] further demonstrated that in raw fermented sausages inoculated with *Latilactobacillus curvatus*, the bacterial population increased significantly from an initial count of 5.25 ± 0.05 log CFU/g (0 day) to 9.6 ± 0.1 log CFU/g (21 day). *Latilactobacillus curvatus* successfully established stable monocultural dominance, becoming the predominant microbial species in the sausage matrix while effectively suppressing competing spoilage microorganisms through competitive exclusion. Casaburi et al. [17] identified that bacteriocins produced by *Latilactobacillus curvatus* exhibited broad-spectrum antimicrobial activity, particularly against foodborne pathogens including Listeria monocytogenes and Bacillus cereus, as well as other Lactobacillus species. This antimicrobial capacity explains the ecological dominance of *Latilactobacillus curvatus* in fermented meat systems, where it simultaneously achieves numerical superiority and creates an inhibitory environment that restricts spoilage microbiota proliferation.

## 5. Conclusions

This study evaluated the effects of lactic acid bacteria (LAB) bioprotective cultures (*Lactilactilactobacillus sakei*, *Pediococcus acidilactici*, and *Latilactobacillus curvatus*) on the quality characteristics, oxidative properties, and microbial community dynamics of pickled pepper rabbit meat during storage. The results demonstrated that all LAB-treated groups exhibited significantly better preservation effects compared to the CK group, with *Latilactobacillus curvatus* showing the most pronounced quality retention. In the later stages of storage, *Latilactobacillus curvatus* significantly reduced pH levels while exhibiting the highest carbonyl content and the lowest TBARS, TVB-N, and sulfhydryl group values, effectively controlling lipid oxidation, protein oxidation, and degradation. Texture analysis confirmed that all treatments maintained acceptable quality parameters. Microbial community analysis further revealed that *Latilactobacillus curvatus* possessed distinct advantages in microbial regulation, with the LC group displaying the lowest bacterial diversity. *Latilactobacillus curvatus* became the dominant strain in pickled pepper rabbit meat, effectively suppressing the growth and proliferation of spoilage-related bacteria. In conclusion, this study demonstrates that *Latilactobacillus curvatus* outperformed other LAB strains in preserving quality parameters, and its application can significantly improve the storage stability of pickled pepper rabbit meat.

## Data Availability

The original contributions presented in the study are included in the article; further inquiries can be directed to the corresponding authors.

## References

[1] Cao R., Wang B., Bai T., Zhu Y., Cheng J., Zhang J. (2024). Structural and functional impacts of glycosylation-induced modifications in rabbit myofibrillar proteins. Int. J. Biol. Macromol..

[2] Xu C., Wang Y., He J., Pan D., Wang H., Zhou G., Cao J. (2020). The Comparative Research of Structural and Textural Characteristics of Six Kinds of Collagen-based Sauce Braised Meat Products. J. Food Sci..

[3] Schnürer J., Magnusson J. (2005). Antifungal lactic acid bacteria as biopreservatives. Trends Food Sci. Technol..

[4] Lücke F.-K. (2000). Utilization of Microbes to Process and Preserve Meat. Meat Sci..

[5] Ben Mrid R., Benmrid B., Hafsa J., Boukcim H., Sobeh M., Yasri A. (2021). Secondary Metabolites as Biostimulant and Bioprotectant Agents: A Review. Sci. Total Environ..

[6] Fitzgerald D.J., Stratford M., Gasson M.J., Ueckert J., Bos A., Narbad A. (2004). Mode of Antimicrobial Action of Vanillin against *Escherichia coli*, *Lactobacillus plantarum* and *Listeria innocua*. J. Appl. Microbiol..

[7] Marcelli V., Osimani A., Aquilanti L. (2024). Research Progress in the Use of Lactic Acid Bacteria as Natural Biopreservatives against *Pseudomonas* spp. in Meat and Meat Products: A Review. Food Res. Int..

[8] Lopez-Arvizu A., Rocha-Mendoza D., Ponce-Alquicira E., García-Cano I. (2021). Characterization of Antibacterial Activity of a N-Acetylmuramoyl-l-Alanine Amidase Produced by *Latilactobacillus sakei* Isolated from Salami. World J. Microbiol. Biotechnol..

[9] Wang Q., Zhang K., Li M., Liu H., Kong B., Chen Q. (2024). Bioprotective Potential of *Latilactobacillus sakei* and *Latilactobacillus curvatus* in Smoked Chicken Legs with Modified Atmosphere Packaging. Food Control.

[10] Muñoz R., de Rivas B.L., Rodríguez H., Esteban-Torres M., Reverón I., Santamaría L., Landete J.M., Plaza-Vinuesa L., Sánchez-Arroyo A., Jiménez N. (2024). Food Phenolics and *Lactiplantibacillus plantarum*. Int. J. Food Microbiol..

[11] Parlindungan E., Lugli G.A., Ventura M., van Sinderen D., Mahony J. (2021). Lactic Acid Bacteria Diversity and Characterization of Probiotic Candidates in Fermented Meats. Foods.

[12] Stadnik J., Kęska P., Gazda P., Siłka Ł., Kołożyn-Krajewska D. (2022). Influence of LAB Fermentation on the Color Stability and Oxidative Changes in Dry-Cured Meat. Appl. Sci..

[13] Wang Z., He Z., Emara A.M., Gan X., Li H. (2019). Effects of Malondialdehyde as a Byproduct of Lipid Oxidation on Protein Oxidation in Rabbit Meat. Food Chem..

[14] Chen Q., Kong B., Han Q., Liu Q., Xu L. (2016). The Role of Bacterial Fermentation in the Hydrolysis and Oxidation of Sarcoplasmic and Myofibrillar Proteins in Harbin Dry Sausages. Meat Sci..

[15] Cha H., Liang S., Shi K., Xu Z., Ge C., Zhao P., Xiao Z. (2023). Effect of Modified Atmosphere Packaging on the Quality Characteristics and Bacterial Community Succession of Super-Chilled Chicken Meat in Biopreservation. LWT.

[16] Lou X., Wen X., Chen L., Shu W., Wang Y., Hoang T.T., Yang H. (2023). Changes in Texture, Rheology and Volatile Compounds of Golden Pomfret Sticks Inoculated with *Shewanella baltica* during Spoilage. Food Chem..

[17] Casaburi A., Martino V.D., Ferranti P., Picariello L., Villani F. (2016). Technological Properties and Bacteriocins Production by *Lactobacillus curvatus* 54M16 and Its Use as Starter Culture for Fermented Sausage Manufacture. Food Control.

[18] Feranchuk S., Belkova N., Potapova U., Kuzmin D., Belikov S. (2018). Evaluating the Use of Diversity Indices to Distinguish between Microbial Communities with Different Traits. Res. Microbiol..

[19] Radziejewska-Kubzdela E., Czaczyk K. (2016). The Effect of Organic Acid Pretreatment and Modified Atmosphere on Shelf Life of Dry Coleslaw Mix. J. Food Process. Preserv..

[20] Rodrigues S.S.Q., Leite A., Vasconcelos L., Teixeira A. (2024). Exploring the Nexus of Feeding and Processing: Implications for Meat Quality and Sensory Perception. Foods.

[21] Cao X., Zhao M., Zou S., Li Z., Wu Y., Ji C., Chen Y., Dong L., Zhang S., Liang H. (2022). Effect of Autochthonous Lactic Acid Bacteria-Enhanced Fermentation on the Quality of Suancai. Foods.

[22] Mejri L., Ziadi A., El Adab S., Boulares M., Essid I., Hassouna M. (2017). Effect of Commercial Starter Cultures on Physicochemical, Microbiological and Textural Characteristics of a Traditional Dry Fermented Sausage Reformulated with Camel Meat and Hump Fat. J. Food Meas. Charact..

[23] Dai S.-Y., Wang P.-M., Lin K.-W. (2021). Application of Lactic Acid Bacteria Isolates from Sugary Kefir Grains to Fermented Semi-Dry Sausage. J. Food Process. Preserv..

[24] Shang H., Yue Y., Guo B., Ji C., Zhang S., Dong L., Ferrocino I., Cocolin L.S., Lin X. (2024). The Effects of *Lactiplantibacillus plantarum* 3-19 and *Pediococcus pentosaceus* 18-1 on Preventing the Accumulation of Biogenic Amines and Promoting the Production of Volatile Organic Compounds during Sour Meat Fermentation. Int. J. Food Microbiol..

[25] Narasimulu J., Baburajan N., Saravanan T.S., Raorane C.J., Vaidyanathan V.K., Ravichandran V., Rajasekharan S.K. (2024). Dipeptides from *Lactiplantibacillus plantarum* Limit *Pseudomonas aeruginosa* Pathogenesis. J. Appl. Microbiol..

[26] Tatiyaborworntham N., Oz F., Richards M.P., Wu H. (2022). Paradoxical effects of lipolysis on the lipid oxidation in meat and meat products. Food Chem. X.

[27] Reitznerová A., Šuleková M., Nagy J., Marcinčák S., Semjon B., Čertík M., Klempová T. (2017). Lipid Peroxidation Process in Meat and Meat Products: A Comparison Study of Malondialdehyde Determination between Modified 2-Thiobarbituric Acid Spectrophotometric Method and Reverse-Phase High-Performance Liquid Chromatography. Molecules.

[28] Gao F., Zhang K., Wang D., Xia L., Gu Y., Tian J., Jin Y. (2024). Effect of Lactobacillus Helveticus IMAUJBH1 on Fat and Volatile Flavor Substances in Fermented Mutton Sausages. Food Chem. X.

[29] Fıçıcılar B.B., Gençcelep H. (2021). Influence of Nisin and Lysozyme on the Shelf Life of Hot-Smoked Rainbow Trout Fillets (*Oncorhynchus mykiss*) during Storage at 4 °C. Philipp. J. Sci..

[30] Zhang Y., Hu P., Lou L., Zhan J., Fan M., Li D., Liao Q. (2017). Antioxidant Activities of Lactic Acid Bacteria for Quality Improvement of Fermented Sausage. J. Food Sci..

[31] Chen Q., Kong B., Han Q., Xia X., Xu L. (2017). The Role of Bacterial Fermentation in Lipolysis and Lipid Oxidation in Harbin Dry Sausages and Its Flavour Development. LWT-Food Sci. Technol..

[32] Bai T., Wang X., Du W., Cheng J., Zhang J., Zhang Y., Klinjapo R., Asavasanti S., Yasurin P. (2025). Recent Advances, Challenges, and Functional Applications of Natural Phenolic Compounds in the Meat Products Industry. Antioxidants.

[33] Estévez M., Díaz-Velasco S., Martínez R. (2021). Protein Carbonylation in Food and Nutrition: A Concise Update. Amino Acids.

[34] Estévez M. (2011). Protein carbonyls in meat systems: A review. Meat Sci..

[35] Al-Sahlany S.T.G., Niamah A.K. (2022). Bacterial Viability, Antioxidant Stability, Antimutagenicity and Sensory Properties of Onion Types Fermentation by Using Probiotic Starter during Storage. Nutr. Food Sci..

[36] Li Z., Su W., Mu Y., Qi Q., Jiang L. (2024). Effects of *Pediococcus acidilactici* and *Rhizopus oryzae* on Protein Degradation and Flavor Formation in Fermented Mutton Sausages. LWT.

[37] Berardo A., Claeys E., Vossen E., Leroy F., Smet S.D. (2015). Protein oxidation affects proteolysis in a meat model system. Meat Sci..

[38] Groussard C., Morel I., Chevanne M., Monnier M., Cillard J., Delamarche A. (2000). Free radical scavenging and antioxidant effects of lactate ion: An in vitro study. J. Appl. Physiol..

[39] Bryukhanov A.L., Klimko A.I., Netrusov A.I. (2022). Antioxidant properties of lactic acid bacteria. Microbiology.

[40] Takeda S., Matsufuji H., Nakade K., Takenoyama S.-I., Ahhmed A., Sakata R., Kawahara S., Muguruma M. (2016). Investigation of Lactic Acid Bacterial Strains for Meat Fermentation and the Product’s Antioxidant and Angiotensin-I-Converting-Enzyme Inhibitory Activities. Anim. Sci. J..

[41] Guo Z., Han L., Yu Q., Lin L. (2020). Effect of a Sea Buckthorn Pomace Extract-Esterified Potato Starch Film on the Quality and Spoilage Bacteria of Beef Jerky Sold in Supermarket. Food Chem..

[42] Bekhit A.E.-D.A., Holman B.W.B., Giteru S.G., Hopkins D.L. (2021). Total volatile basic nitrogen (TVB-N) and its role in meat spoilage: A review. Trends Food Sci. Technol..

[43] Li J., Yang X., Shi G., Chang J., Liu Z., Zeng M. (2019). Cooperation of Lactic Acid Bacteria Regulated by the AI-2/LuxS System Involve in the Biopreservation of Refrigerated Shrimp. Food Res. Int..

[44] Sun F., Kong B., Chen Q., Han Q., Diao X. (2017). *N*-Nitrosoamine Inhibition and Quality Preservation of Harbin Dry Sausages by Inoculated with *Lactobacillus pentosus*, *Lactobacillus curvatus* and *Lactobacillus sake*. Food Control.

[45] Hilbig J., Hildebrandt L., Herrmann K., Weiss J., Loeffler M. (2020). Influence of Homopolysaccharide-producing Lactic Acid Bacteria on the Spreadability of Raw Fermented Sausages (Onion Mettwurst). J. Food Sci..

[46] Jin G., Zhang M., Wang X., Zhang Y., Jiang G., Mei L. (2025). Characteristics of Exopolysaccharides-Egg White Protein Composite Gel and Its Application in Low-Fat Sausage. Food Chem..

[47] Riebroy S., Benjakul S., Visessanguan W. (2008). Properties and Acceptability of Som-Fug, a Thai Fermented Fish Mince, Inoculated with Lactic Acid Bacteria Starters. LWT-Food Sci. Technol..

[48] Slima S.B., Ktari N., Trabelsi I., Triki M., Feki-Tounsi M., Moussa H., Makni I., Herrero A., Jiménez-Colmenero F., Perez C.R.-C. (2017). Effect of partial replacement of nitrite with a novel probiotic *Lactobacillus plantarum* TN8 on color, physico-chemical, texture and microbiological properties of beef sausages. LWT-Food Sci. Technol..

[49] Trabelsi I., Slima S.B., Ktari N., Triki M., Abdehedi R., Abaza W., Moussa H., Abdeslam A., Salah R.B. (2019). Incorporation of Probiotic Strain in Raw Minced Beef Meat: Study of Textural Modification, Lipid and Protein Oxidation and Color Parameters during Refrigerated Storage. Meat Sci..

[50] Wang Y., Han J., Wang D., Gao F., Zhang K., Tian J., Jin Y. (2022). Research Update on the Impact of Lactic Acid Bacteria on the Substance Metabolism, Flavor, and Quality Characteristics of Fermented Meat Products. Foods.

[51] Castellano P., González C., Carduza F., Vignolo G. (2010). Protective Action of *Lactobacillus curvatus* CRL705 on Vacuum-Packaged Raw Beef. Effect on Sensory and Structural Characteristics. Meat Sci..

[52] Xu M.M., Kaur M., Pillidge C.J., Torley P.J. (2021). Evaluation of the Potential of Protective Cultures to Extend the Microbial Shelf-Life of Chilled Lamb Meat. Meat Sci..

[53] Shao X., Wang H., Song X., Xu N., Sun J., Xu X. (2024). Effects of Different Mixed Starter Cultures on Microbial Communities, Taste and Aroma Compounds of Traditional Chinese Fermented Sausages. Food Chem. X.

[54] Liu Y., Chen X., Li F., Shi H., He M., Ge J., Ling H., Cheng K. (2023). Analysis of Microbial Diversity and Metabolites in Sauerkraut Products with and without Microorganism Addition. Foods.

[55] Qiu Z., Li N., Lu X., Zheng Z., Zhang M., Qiao X. (2018). Characterization of Microbial Community Structure and Metabolic Potential Using Illumina MiSeq Platform during the Black Garlic Processing. Food Res. Int..

[56] Gajendran V.P., Rajamani S. (2024). Recent Advancements in Harnessing Lactic Acid Bacterial Metabolites for Fruits and Vegetables Preservation. Probiotics Antimicrob. Proteins.

[57] Janßen D., Ehrmann M.A., Vogel R.F. (2019). Monitoring of Assertive *Lactobacillus sakei* and *Lactobacillus curvatus* Strains Using an Industrial Ring Trial Experiment. J. Appl. Microbiol..

